# Impact of Multidrug-Resistant Bacteria in a Cohort of COVID-19 Critically Ill Patients: Data from a Prospective Observational Study Conducted in a High-Antimicrobial-Resistance-Prevalence Center

**DOI:** 10.3390/jcm14020410

**Published:** 2025-01-10

**Authors:** Giorgia Montrucchio, Francesca Grillo, Eleonora Balzani, Giulia Gavanna, Gabriele Sales, Chiara Bonetto, Umberto Simonetti, Marinella Zanierato, Vito Fanelli, Claudia Filippini, Silvia Corcione, Francesco Giuseppe De Rosa, Antonio Curtoni, Cristina Costa, Luca Brazzi

**Affiliations:** 1 Department of Surgical Sciences, University of Turin, 10126 Turin, Italy; francesca.grillo@unito.it (F.G.); eleonora.balzani@unito.it (E.B.); giulia.gavanna@edu.unito.it (G.G.); g.sales@unito.it (G.S.); vito.fanelli@unito.it (V.F.); claudia.filippini@unito.it (C.F.); luca.brazzi@unito.it (L.B.); 2 Department of Anesthesia, Intensive Care and Emergency, Città della Salute e della Scienza Hospital, 10126 Turin, Italy; cbonetto2@cittadellasalute.to.it (C.B.); usimonetti@cittadellasalute.to.it (U.S.); mzanierato@cittadellasalute.to.it (M.Z.); 3 Department of Medical Sciences, University of Turin, 10124 Turin, Italy; silvia.corcione@unito.it (S.C.); francescogiuseppe.derosa@unito.it (F.G.D.R.); 4 Division of Geographic Medicine and Infectious Diseases, Tufts University School of Medicine, Boston, MA 02111, USA; 5 Department of Public Health and Paediatrics, University of Turin, 10124 Turin, Italy; antonio.curtoni@unito.it (A.C.); cristina.costa@unito.it (C.C.); 6 Microbiology and Virology Laboratory, Città Della Salute e Della Scienza Hospital, Corso Dogliotti 14, 10126 Turin, Italy

**Keywords:** COVID-19, intensive care unit, superinfection, multidrug-resistant organism, *Acinetobacter baumannii*, *Klebsiella pneumoniae*, antimicrobial resistance

## Abstract

**Background**: Bacterial superinfections are common complications during viral infections, but the impact of multidrug-resistant (MDR) pathogens in critically ill patients affected by coronavirus disease 2019 (COVID-19) is still debated. **Methods**: This is an observational, monocentric, and prospective study designed to investigate the incidence, risk factors, and outcomes of MDR bacterial superinfections in COVID-19 patients admitted to the intensive care unit (ICU). **Results**: A high incidence of superinfections (66%, 159/241) was observed: ventilator-associated pneumonia (VAP) (65%, 104/159) and bloodstream infection (BSI, 32%, 51/159) were the most common. Superinfections, Extra-Corporeal Membrane Oxygenation (ECMO) support, and prone positioning increased the risk of death five, four, and more-than-two times, respectively (OR = 5.431, IC 95%: 1.637–18.014; 4.462, IC 95%: 1.616–12.324 and 2.346, IC 95%: 1.127–4.883). MDR bacteria were identified in 61% of patients with superinfection, with a cumulative incidence of 37.2% at day 14. Carbapenem-resistant *Acinetobacter baumannii* (CR-AB) and CR-*Klebsiella pneumoniae* (CR-KP) were the most common causative agents (24.3% and 13.7%). CR-AB was found to significantly increase both ICU and in-hospital mortality (76.4% and 78.2%), whereas CR-KP had no direct impact on mortality. Prior rectal colonization (*p* < 0.0001), mechanical ventilation (*p* = 0.0017), a prolonged ICU stay (*p* < 0.0001), the use of iNO (*p* = 0.0082), vasopressors (*p* = 0.0025), curarization (*p* = 0.0004), and prone positioning (*p* = 0.0084) were found to be risk factors for CR-AB. **Conclusions**: Critically ill COVID-19 patients are at high risk of developing MDR superinfection. While CR-KP had no direct impact on mortality, CR-AB appeared to increase ICU and in-hospital mortality.

## 1. Introduction

Bacterial and fungal superinfections are common in patients presenting with viral infections and they are typically associated with a poor outcome. Critically ill patients with SARS-CoV-2 are at high risk of developing superinfections, due to the immune suppression associated with the SARS-CoV-2 infection. In addition, the use of immunomodulators, the frequent need for intensive care unit (ICU) admission, and the use of invasive life-support procedures significantly increase the risk of multidrug-resistant (MDR)-related infections in this cohort. A growing body of evidence confirms the role of superinfections as a leading cause of mortality among COVID-19 patients, suggesting an association between the risk of infection and COVID-19 severity [1,2].

While the role of MDR bacteria might have been underestimated, its importance is growing due to the complex interplay of several factors, such as the number of ICU admissions, the heavy workload, the use of personal protective equipment, and staff/ward relocation. Indeed, in many settings the local ecology has been strongly influenced by the growing burden of antimicrobial resistance (AMR) [3], which was already affecting healthcare systems and countries, such as Italy, before the pandemic [4]. Among the infectious agents reported to be of particular importance we can mention Gram-negative bacteria (GNB), in particular extended-spectrum beta-lactamase (ESBL) and carbapenem-resistant bacteria. The consecutive epidemiological waves of the COVID-19 pandemic have undoubtedly facilitated the spread of AMR. Reasons for this growth include the increased use of broad-spectrum antimicrobials, a high turnover of ICU admissions, an increased number of patients requiring high-intensity care (endotracheal intubation and venovenous Extra-Corporeal Membrane Oxygenation [vv-ECMO] treatment), and limited adherence to antimicrobial stewardship and infection control measures.

The aim of this study was to evaluate the incidence, clinical and microbiological characteristics, risk factors, and outcomes of superinfections in a large cohort of COVID-19 patients admitted to the ICU between March 2020 and December 2021. We also evaluated, in a context of high pre-pandemic MDR incidence [5,6], the impact of MDR-related superinfections, with a focus on GNB, especially *Acinetobacter baumannii* and carbapenem-resistant Klebsiella pneumoniae.

## 2. Materials and Methods

### 2.1. Study Design and Setting

This is a prospective observational study conducted between March 2020 and December 2021 in the referral intensive care unit for the ECMO support of the Piedmont region (Italy) of the “Città della Salute e della Scienza” University Hospital in Turin, Italy, and the temporary ICU unit, activated in the same hospital, during the pandemic. The available beds in the two ICUs were 10 and 8, respectively, with 3 isolation units in total.

This study was designed and implemented in accordance with the principles stated in the Declaration of Helsinki and was approved by the Local Ethics Committee (Study Number 0069865, dated 21 July 2020). The data collection process was conducted anonymously, and the need for individual consent was waived by the Local Ethics Committee.

### 2.2. Patients’ Management

All adult patients diagnosed with COVID-19-related pneumonia, confirmed by a Reverse Transcriptase Polymerase Chain Reaction (RT-PCR) test on a lower respiratory tract specimen [7], were included.

Patients were treated according to internal protocols for the management of severe respiratory failure, incorporating the latest guidelines derived from COVID-19 literature [8], and were considered eligible for venovenous Extra-Corporeal Membrane Oxygenation (vv-ECMO) respiratory support according to international criteria [9]. Closed lung suction systems were used.

Routine surveillance cultures (endotracheal aspirate, rectal swabs for MDR bacteria) were obtained at ICU admission and weekly thereafter; additional tests (e.g., blood culture, bronchoalveolar lavage [BAL], bronchial aspirate [BA], and the detection of respiratory viruses) were performed in the presence of a clinical or laboratory suspicion of infection, according to the discretion of the attending intensivists.

### 2.3. Data Collection

The following data were collected: demographic characteristics, comorbidities, Charlson comorbidity index, Sequential Organ Failure Assessment (SOFA) and Simplified Acute Physiology Score II (SAPS II) scores, site of sample collection and method (endotracheal aspirate [ETA], BA, BAL, blood culture, or other cultures), bacterial species, antibiotic susceptibility, ICU and hospital mortality, and length of ICU and hospital stay.

All episodes of ventilator-associated pneumonia (VAP), bloodstream infection (BSI), the need for vasopressor therapy, and the presence of septic shock were also registered.

### 2.4. Definitions

Bacterial superinfection was defined as clinical deterioration and the presence of bacteria identified in the lower respiratory tract (ETA, BA, or BAL), urine culture, blood culture, or other culture samples (e.g., pleural effusion, ascitic fluid) after 48 h of ICU admission.

VAP and BSI were defined according to the current ECDC definitions [10,11].

MDR pathogens were defined as all pathogens resistant to at least one agent in three or more antimicrobial classes of agents, according to Magiorakos and colleagues [12].

### 2.5. Statistical Analysis

Summary data are presented as means and standard deviations (SD) or medians and interquartile ranges (IQR) for continuous variables; numbers and percentages are reported for categorical variables. In the univariate analysis, continuous variables were compared using the Wilcoxon–Mann–Whitney test. Categorical variables were compared using Fisher’s exact test or the Chi-square test, as appropriate.

Univariate analyses stratified by both superinfection and survival were conducted. The Kaplan–Meier method considering a period of 30 days was used for survival analysis.

A Log-rank test was used to assess the differences between survival curves, considering the presence of superinfections and addressing each infection type (i.e., VAP, BSI).

A logistic regression model adjusted for age, diabetes mellitus, cardiovascular disease, chronic lung disease, SOFA and MuLBSTA score at admission, neutrophil count, lymphocyte count and lactate at admission, D-dimer and pro-ADM measured within 48 h of ICU admission, presence of superinfections, MV, iNO, ECMO during ICU stay, RRT, and prone positioning was used to test potential confounding factors on mortality. All results are presented as odds ratios (ORs) and confidence intervals (CIs) at the 95% level.

A multivariate logistic regression analysis for superinfection adjusted for age, gender, SOFA and MuLBSTA scores at ICU admission, lactate concentration at admission, ECMO support at admission, and prone positioning was also used.

All analyses were two-sided with *p*-values less than 0.05 deemed statistically significant. All analyses were performed with the open-source R (R project version 3.5.0) and SAS software, version 9.4 (SAS Institute Inc., Cary, NC, USA).

## 3. Results

### 3.1. Studied Population

A total of 241 patients were enrolled (Table 1). Only diabetes mellitus was found to significantly increase mortality (*p*-value = 0.0325), while elevated SOFA, MuLBSTA, and SAPS II values at ICU admission and the use of invasive procedures (invasive mechanical ventilation [IMV], curarization, prone positioning, inhaled nitric oxide [iNO], renal replacement therapy [RRT], ECMO, and vasoactive drugs) were found to be associated with a worse outcome.

Fifty-two (21.6%) patients underwent ECMO treatment (44 [18.3%] at ICU admission and 52 [21.6%] during ICU stay) and showed an increased risk of mortality (*p*-value = 0.0013 and <0.0001, respectively).

ICU and in-hospital mortality rates were 55% (n = 133) and 60% (n = 145), respectively. The median length of ICU stay was 10 (6–22) and 18 (13–26) days among survivors and among non-survivors, respectively (*p*-value = 0.0035).

Pre-ICU, ICU, and hospital length of stay were significantly associated with mortality (*p*-value = 0.0013, 0.0035, and 0.0016, respectively).

Thirty-three (13.7%) patients had an infectious complication within 48 h of ICU admission (co-infection), 159 (66%) developed a superinfection during ICU stay, and both groups had a significantly higher mortality compared to non-infected patients (*p*-value < 0.0025 and <0.0001, respectively). Septic shock, documented in 73 (30.3%) superinfected patients, was found to significantly increase mortality (*p*-value < 0.0001).

The multivariate logistic regression analysis showed that mortality was independently associated with the following: superinfection (OR = 5.431, IC 95%: 1.637–18.014; *p*-value = 0.0057), ECMO (OR = 4.462, IC 95%: 1.616–12.324, *p*-value = 0.0039), prone positioning (OR = 2.346, IC 95%: 1.127–4.883, *p*-value = 0.0226), a higher MuLBSTA score (OR = 1.173, IC 95%: 1.058–1.300, *p*-value = 0.0024), and a higher SOFA score (OR = 1.137, IC 95%: 1.016–1.273, *p*-value = 0.0253).

### 3.2. Effects of Superinfection

One hundred fifty-nine (66%) patients had a secondary infection during the ICU stay (Table 2). A higher lactate concentration at ICU admission (*p*-value = 0.0208), higher SOFA (*p*-value = 0.0160) and MuLBSTA (*p*-value = 0.0018) scores, and the use of invasive procedures, namely MV (*p*-value < 0.0001), prone positioning (*p*-value = 0.0010), iNO (*p*-value < 0.0001), RRT (*p*-value = 0.0053), ECMO (*p*-value = 0.0004), and the use of vasopressor therapy (*p*-value < 0.0001), were all associated with the development of superinfection. The use of steroids was also found to be associated with a higher risk of secondary infections (*p*-value < 0.0161), whereas no association was observed between the use of immunosuppressive drugs (*p*-value = 0.6172) or tocilizumab (*p*-value = 0.8667) and secondary infections. Rectal colonization by MDR bacteria also appeared to be significantly associated with the occurrence of superinfection (*p*-value < 0.0001).

Both ICU and in-hospital mortality were higher among superinfected patients (68% and 73%, respectively) compared with non-superinfected patients (31% and 35%, respectively; *p*-value < 0.0001 and <0.0001).

Patients who experienced infectious complications had a significantly longer duration of ICU and hospital stay (median of 18 [IQR 12–27] days and 25 [IQR 18–34] days versus 8 [IQR 5–11] days and 19 [IQR 13–31] days, respectively; *p*-value < 0.0001 and 0.0027, respectively).

The multivariate logistic regression analysis for superinfection adjusted for age, gender, SOFA and MuLBSTA scores and lactate concentration at ICU admission, ECMO support at admission, and prone positioning showed a statistically significant odds ratio, equal to 1.161 [95% CI: 1.048–1.287] (*p*-value = 0.0043) for the MuLBSTA score, 3.446 [95% CI: 1.157–10.266] (*p*-value = 0.0263) for ECMO, and equal to 2.555 [95% CI: 1.110–4.578] (*p*-value = 0.0245) for prone positioning.

VAP was the most common secondary infection (104/159 [65%]), followed by BSI (51/159 [32%]) and abdominal infection (4/159 [2.5%]). Septic shock was documented in 75 out of 159 patients (46%).

MDR pathogens (specifically carbapenem-resistant *Acinetobacter baumannii* and carbapenem-resistant *Klebsiella pneumoniae*) were the causative agent in most cases (24.3% and 13.7% of isolates, respectively), followed by *Staphylococcus aureus* (14.4% of isolates, mainly methicillin-susceptible *Staphylococcus aureus*, which accounted for 9.5% of isolates), *Enterococci* (10.3% of isolates), and coagulase-negative methicillin-resistant *Staphylococci* (6.8% of isolates) (Table 3).

Among patients with superinfection, 126 (126/159, 79.2%) had infectious complications resulting from Gram-negative bacteria (GNB), mainly carbapenem-resistant *Acinetobacter baumannii* (64 isolates, 37.9%) and carbapenem-resistant *Klebsiella pneumoniae* (36 isolates, 21.3%, out of a total of 169 isolated GNB) (Table 3).

### 3.3. Effects of Multidrug-Resistant Bacteria (MDR) Superinfection

Among patients with superinfection, 97 (61%) demonstrated at least one infectious complication resulting from MDR bacteria (Gram-negative and/or Gram-positive). The cumulative incidence was 17.1%, 37.2%, and 47% at day 7, 14, and 21, respectively (Table 4).

The use of iNO and vasopressors was significantly higher in MDR superinfected patients (33% and 86.6%, respectively, *p*-value = 0.0003), compared to non-MDR superinfected patients (12.8% and 58%, respectively, *p*-value < 0.0001), as was the use of steroids (80.4% versus 67.4%, *p*-value = 0.0130). Patients superinfected with MDR bacteria required IMV more frequently during the ICU course (96% versus 83%, *p*-value < 0.0001), with a median of 18 (IQR 13–25) days in the MDR group and 8 (IQR 5–16) days in the non-MDR group (*p*-value < 0.0001). The proportion of patients undergoing ECMO support was also significantly higher in the MDR population (32%) compared to the non-MDR population (14.2%, *p*-value 0.0020).

The ICU stay was significantly longer for MDR superinfected patients (median of 20 days [IQR 14–29]) compared to non-MDR superinfected patients (median of 10 days [IQR 6–17], *p*-value < 0.0001). The MDR group had higher ICU and in-hospital mortality rates (70% and 72%, versus 44% and 51%, respectively, *p*-value < 0.0001 and 0.0012).

VAP was the most common superinfection, and 70.3% of VAP cases were the result of MDR bacteria. Rectal colonization by carbapenem-resistant MDR bacteria was more common in MDR superinfected patients (82.5% versus 34.8%, *p*-value < 0.0001). In addition, MDR superinfected patients were more frequently exposed to carbapenems (23.7% versus 12%, *p*-value = 0.0208).

### 3.4. Effects of Carbapenem-Resistant Klebsiella Pneumoniae (CR-KP) Superinfection

Among patients with superinfection, 38 (23.9%) developed at least one infectious complication resulting from carbapenem-resistant *Klebsiella pneumoniae* (CR-KP) (Table 5).

This subgroup was characterized by the following:

- A longer duration of mechanical ventilation (median of 23 [IQR 14–34] days vs. 12 [IQR 6–19], *p*-value < 0.0001);
- A higher proportion of patients undergoing ECMO support (42% vs. 17.5%, *p*-value 0.0020);
- A longer ICU and hospital stay (median of 23 days [IQR 16–35] vs. 13 [IQR 7–20], *p*-value < 0.0001, and 31 days [IQR 24–46] vs. 22 [IQR 15–32], *p*-value < 0.0001, respectively).

No difference concerning either ICU or in-hospital mortality was observed between the two subgroups (*p*-value < 0.2885 and 0.4724, respectively).

### 3.5. Effects of Carbapenem-Resistant Acinetobacter baumannii (CR-AB) Superinfection

Among patients with superinfection, 55 (34.6%) developed at least one secondary infection resulting from carbapenem-resistant *Acinetobacter baumannii* (CR-AB) (Table 6).

This subgroup was characterized by the following:

- A greater use of iNO and vasoactive drugs (34.5% and 89% vs. 16.9% and 64%, *p*-value 0.0082 and 0.0025, respectively);
- A greater proportion of patients requiring prone positioning and curarization (87.3% and 100% vs. 67.8% and 79%, *p*-value 0.0084 and 0.0004, respectively);
- A greater proportion of patients treated with mechanical ventilation during the ICU stay (98.2% vs. 85.2%, *p*-value 0.0017);
- A higher frequency of septic shock (43.6% vs. 25%, *p*-value 0.0178);
- A longer ICU stay (median 18 days [IQR 14–24] vs. 12 [IQR 7–22], *p*-value < 0.0001) with a similar length of hospital stay (*p*-value 0.0918);
- Higher ICU and in-hospital mortality rates (76.4% vs. 48%, *p*-value 0.0002, and 78.2% vs. 54%, *p*-value 0.0016), respectively.

## 4. Discussion

While at the beginning of the pandemic the issue of superinfections in patients with COVID-19 might have been underestimated, it soon became clear that it greatly affects the outcome of the most severely ill patients [5]. However, although several studies reported data on COVID-19 patients, most of them have failed to define the real impact of superinfections caused by MDR pathogens on this population.

The present prospective observational study conducted on 241 critically ill COVID-19 patients seems to highlight that, at least in this population, superinfections are more frequent (66%) compared to what has been reported in the literature [1,2,13,14]. The presence of superinfection increased the risk of death five times, the need for ECMO support increased the risk of mortality fourfold, and the need for prone positioning more than doubled the risk. Among other variables independently associated with an increased risk of mortality was a higher MuLBSTA score.

In our study, 13.7% of patients experienced an infectious complication within 48 h from ICU admission (i.e., co-infections). Superinfected patients showed higher mortality rates (68% in the ICU and 73% in the hospital) compared to the pre-pandemic era [15] and to the “first wave” of the pandemic [13,16]. VAP was the most common superinfection (65%, 104/159), followed by BSI (32%, 51/159): these data are in line with previous literature findings [17,18] and highlight that VAP is closely related to severe respiratory conditions in COVID-19 patients. Among patients undergoing ECMO therapy, 86.5% developed a superinfection during the ICU course, confirming that invasive life-support procedures and the severity of the respiratory disease are associated with an increased risk of developing secondary infections.

Risk factors that were found to favor the development of superinfections in our cohort were as follows: a higher lactate concentration at ICU admission, higher SOFA and MuLBSTA scores, MV, prone positioning, iNO, RRT, ECMO, the use of vasopressor therapy and steroids, and enteric colonization by MDR bacteria. Furthermore, when evaluating the specific impact of MDR pathogens on COVID-19 patients’ outcome, some differences emerged when considering specific microorganisms and specific resistance patterns: while CR-AB was associated with a worse outcome, CR-KP had no direct impact on mortality.

The possible role of immunomodulatory/immunosuppressive therapy is debatable. In a retrospective cohort study, Guaraldi and colleagues reported that tocilizumab was associated with an increased risk of superinfection [19]. Nevertheless, we found no association between the occurrence of secondary infections and the use of tocilizumab, possibly due to the small number of patients who received this treatment and the fact that tocilizumab was administered as a single dose.

Our study showed a predominance of MDR Gram-negative bacteria (*Acinetobacter baumannii* and *Klebsiella pneumoniae*), in line with data reported for Italy by the European Centre for Disease Prevention and Control [3], followed by methicillin-susceptible *Staphylococcus aureus*, *Enterococci*, and coagulase-negative methicillin-resistant *Staphylococci*.

A higher incidence of MDR pathogens compared to European and Italian reports had already been documented in our ICU during the pre-pandemic era [15,20,21] and this pattern was also confirmed in the early stages of the pandemic [5,22]. An increased prevalence of *Acinetobacter baumannii* was also highlighted [6].

The present study identified MDR bacteria in 61% of all patients with bacterial superinfection.

The inappropriate use of personal protective equipment and suboptimal adherence to standard infection control policies have been described as major factors contributing to the outbreak of nosocomial MDR infections during the pandemic period. Causal factors might be the increased need to handle each patient, with multiple patients during the same shift, for pronation and the required positional changes, to verify correct positioning, and the devices in use; the overlap between isolation measures for COVID-19 and contact measures with greater procedural difficulties; and, at the beginning, the poor availability of personal protective equipment. This seems to be confirmed by the finding of prone position as a risk factor for the development of MDR superinfection [6].

MDR pathogens had a huge impact on both ICU and in-hospital mortality: among patients with MDR superinfection, 70% died in the ICU and 72% in the hospital.

The cumulative incidence of MDR superinfection was 37.2% at day 14: this finding underlines the need to pursue antimicrobial stewardship principles and infection control activities aimed at combating the spread of antimicrobial resistance. The use of carbapenems appears to be particularly impactful, as 57.5% of patients receiving carbapenems became MDR-superinfected. The misuse/overuse of such molecules leads to the selection of MDR microbes (particularly in the ICU) that negatively impact humans [23].

Regarding CR-KP superinfections, we did not find any statistically significant association between ICU and in-hospital mortality rates: this could be explained by the fact that patients were more likely to develop a CR-KP infection at a later stage, secondary to gastrointestinal colonization.

CR-AB superinfection was found to significantly increase both ICU and in-hospital mortality.

Among the most frequently reported risk factors for CR-AB infections [24,25,26], our data seem to confirm the previous literature, highlighting the role of prior rectal colonization, the need for mechanical ventilation and prone positioning, and a prolonged ICU stay. However, no association was observed between the occurrence of CR-AB superinfection and the use of steroids, possibly due to the timing of administration.

The present study has limitations. First, it is a single-center experience in a population with high disease severity, which may limit the generalizability of our results. Furthermore, the high mortality rates observed in the study population could be partially attributable to patient severity. Second, frequent transfers of patients between hospitals and the initial suboptimal adherence to infection control policies might have contributed to the selection of pathogens with different profiles of resistance. Another limitation is the long duration of our study, which introduced some heterogeneity in clinical management, such as the use of different immunomodulatory, antibiotic, and antiviral therapies that unfortunately we were not able to collect and analyze. Finally, the high incidence of MDR microorganisms in our center in the pre-pandemic phase has certainly influenced our findings.

## 5. Conclusions

Critically ill COVID-19 patients are at high risk of developing superinfections caused by MDR microorganisms, with a concomitant increase in morbidity and mortality. While CR-KP had no direct impact on mortality, CR-AB appeared to increase both ICU and in-hospital mortality.

The impact of MDR Gram-negative invasive bacterial infections on mortality should be kept in mind as it reiterates the importance of considering the hospital microbiological ecology and applying effective and rigorous infection control policies (hand hygiene, patient cohorting, epidemiological surveillance systems) and antimicrobial stewardship programs.

## Figures and Tables

**Table 1:** Baseline characteristics among survivors and non-survivors.

Variable	Total (*N* = 241)	Survivors (*N* = 108)	Non-Survivors (*N* = 133)	*p*-Value
Age, years, mean ± SD	63.7 ± 11.4	62.0 ± 12.3	65.0 ± 10.4	0.0835
Gender, male, n (%)	180 (74.7)	80 (74.1)	100 (75.2)	0.8822
BMI, kg/m^2^, median (IQR)		28.3 (25.4–31.5)	27.8 (25.6–31.2)	0.8228
Lactate concentration, mmol/L, mean ± SD		1.69 ± 1.72	2.23 ± 1.70	<0.0001
SOFA at admission, median (IQR)	7 (4–9)	6 (4–8)	8 (5–10)	<0.0001
MuLBSTA at admission, median (IQR)	11 (9–13)	11 (7–13)	13 (9–15)	<0.0001
SAPS II at admission, median (IQR)	48 (40–58)	44 (39–56)	54 (43–59)	0.0043
Patients with ≥1 comorbidity, n (%)	216 (89.6)	93 (86.1)	123 (92.5)	0.1371
Patients with ≥3 comorbidity, n (%)	117 (48.6)	49 (45.4)	68 (51.1)	0.2316
Obesity, n (%)	129 (53.5)	58 (54.2)	71 (53.8)	1
Hypertension, n (%)	151 (62.7)	62 (57.4)	89 (66.9)	0.1421
Cardiovascular disease, n (%)	46 (19.1)	15 (13.9)	31 (23.3)	0.0713
Lung disease, n (%)	32 (13.3)	9 (8.3)	23 (17.3)	0.0554
Alcohol/drugs, n (%)	8 (3.3)	5 (4.6)	3 (2.3)	0.4729
Chronic kidney disease, n (%)	19 (7.9)	10 (9.3)	9 (6.8)	0.4831
Liver failure, n (%)	4 (1.7)	1 (0.9)	3 (2.3)	0.6300
Neurological disorder, n (%)	21 (8.7)	10 (9.3)	11 (8.3)	0.8214
Solid active neoplasm, n (%)	5 (2.1)	1 (0.9)	4 (3.0)	0.3834
Hematological active neoplasm, n (%)	8 (3.3)	2 (1.9)	6 (4.6)	0.3008
Autoimmune diseases, n (%)	23 (9.5)	11 (10.2)	12 (9.0)	0.8272
Immunosuppressive therapies, n (%)	18 (7.5)	10 (9.3)	8 (6.0)	0.4608
Diabetes mellitus, n (%)	56 (23.2)	18 (16.7)	38 (28.6)	0.0325
Non-invasive ventilation (CPAP), n (%)	159 (66.0)	66 (63.5)	93 (71.0)	0.2616
Non-invasive ventilation (NIV), n (%)	175 (72.6)	74 (69.8)	101 (77.1)	0.2353
IMV at ICU admission, n (%)	152 (63.1)	67 (62.0)	85 (64.9)	0.8735
NIV at ICU admission, n (%)	28 (11.6)	14 (12.9)	14 (10.7)	
IMV during ICU stay, n (%)	213 (88.4)	87 (82.1)	126 (96.2)	0.0004
IMV days, median (IQR)		9 (5–18)	16 (10–23)	0.0013
Steroids, n (%)	176 (73.0)	74 (71.2)	102 (79.1)	0.1711
Tocilizumab, n (%)	49 (20.3)	21 (19.8)	28 (21.4)	0.8721
Curarization, n (%)	202 (83.8)	79 (77.5)	123 (96.8)	<0.0001
Pronation, n (%)	174 (72.2)	68 (64.2)	106 (80.9)	0.0048
iNO, n (%)	51 (21.2)	9 (8.4)	42 (31.8)	<0.0001
Acute kidney injury, n (%)	46 (19.3)	16 (15.0)	30 (22.9)	0.1393
RRT during ICU stay, n (%)	28 (11.6)	6 (5.6)	22 (16.7)	0.0083
Vasoactive drugs during ICU stay, n (%)	169 (70.1)	55 (53.9)	114 (89.8)	<0.0001
ECMO at ICU admission, n (%)	44 (18.3)	10 (9.3)	34 (25.8)	0.0013
ECMO during ICU stay, n (%)	52 (21.6)	11 (10.2)	41 (31.1)	<0.0001
Co-infection within 48 h, n (%)	33 (13.9)	7 (6.5)	26 (20.0)	0.0025
Superinfection during ICU stay, n (%)	159 (66.0)	51 (47.2)	108 (81.2)	<0.0001
Septic shock, n (%)	73 (30.3)	11 (10.4)	62 (47.7)	<0.0001
**Outcomes**	**Total** **(*N* = 241)**	**Survivors** **(*N* = 108)**	**Non-Survivors ** **(*N* = 133)**	***p*-Value**
ICU mortality, n (%)	133 (55.2)			
Hospital mortality, n (%)	145 (60.2)			
ICU LOS, days, median, (IQR)		10 (6–22)	18 (13–26)	0.0035
Hospital LOS, days, median, (IQR)		28 (17–40)	22 (15–30)	0.0016
Pre-ICU LOS, days, median, (IQR)		2 (1–4)	4 (1–9)	0.0013

List of abbreviations: BMI: Body Mass Index; CPAP: Continuous Positive Airway Pressure; ECMO: Extra-Corporeal Membrane Oxygenation; ICU: intensive care unit; IMV: invasive mechanical ventilation; iNO: inhaled nitric oxide; LOS: length of stay; NIV: non-invasive ventilation; RRT: renal replacement therapy.

**Table 2:** Baseline characteristics among patients with and without superinfection.

Variable	Total (*N* = 241)	Superinfection (*N* = 159)	No Superinfection (*N* = 82)	*p*-Value
Age, years, mean ± SD		63.3 ± 10.9	64.4 ± 12.3	0.3117
Gender, male, n (%)	180 (74.7)	118 (74.2)	62 (75.6)	0.8765
BMI, kg/m^2^, median (IQR)		28 (26–31)	28 (25–31)	0.2617
Lactate concentration, mmol/L, mean ± SD		2.2 ± 2.0	1.6 ± 0.9	0.0208
SOFA at ICU admission, median (IQR)	7 (4–9)	7 (5–9)	6 (4–8)	0.0160
MuLBSTA at ICU admission, median (IQR)	11 (9–13)	12 (9–15)	11 (8–13)	0.0018
SAPS II at ICU admission, median (IQR)	48 (40–58)	51 (42–58)	46 (39–59)	0.4355
Patients with ≥3 comorbidity, n (%)	117 (48.5)	74 (46.5)	43 (52.4)	0.3572
Chronic kidney disease, n (%)	19 (7.9)	12 (7.6)	7 (8.5)	0.8039
Cardiovascular diseases, n (%)	46 (19.1)	29 (18.2)	17 (20.7)	0.7296
Lung disease, n (%)	32 (13.3)	19 (11.9)	13 (15.8)	0.4262
Alcohol/drugs, n (%)	8 (3.3)	7 (4.4)	1 (1.2)	0.2702
Liver failure, n (%)	4 (1.7)	2 (1.3)	2 (2.4)	0.6068
Solid active neoplasm, n (%)	5 (2.1)	4 (2.5)	1 (1.2)	0.6640
Hematological active neoplasm, n (%)	8 (3.3)	3 (1.9)	5 (6.2)	0.1235
Autoimmune diseases, n (%)	23 (9.3)	15 (9.4)	8 (9.8)	1
Diabetes mellitus, n (%)	56 (23.2)	39 (24.5)	17 (20.7)	0.6294
Non-invasive ventilation (CPAP), n (%)	159 (66.0)	105 (68.2)	54 (66.7)	0.8835
Non-invasive ventilation (NIV), n (%)	175 (72.6)	119 (76.3)	56 (69.1)	0.2757
IMV during ICU stay, n (%)	213 (88.4)	150 (96.2)	63 (77.8)	<0.0001
IMV days, median (IQR)		16 (11–24)	7 (4–9)	<0.0001
Steroids, n (%)	176 (73.0)	124 (80.5)	52 (65.8)	0.0161
Tocilizumab, n (%)	49 (20.3)	33 (21.2)	16 (19.8)	0.8667
Immunosuppressive therapies, n (%)	18 (7.5)	11 (6.9)	7 (8.5)	0.6172
Pronation, n (%)	174 (72.2)	127 (80.4)	47 (59.5)	0.0010
iNO, n (%)	51 (21.2)	48 (30.4)	3 (3.7)	<0.0001
Acute kidney injury, n (%)	46 (19.1)	31 (19.8)	15 (18.5)	0.8642
RRT during ICU stay, n (%)	28 (11.7)	25 (15.7)	3 (3.7)	0.0053
Days of RRT, median (IQR)		6 (2–14)	1 (1–7)	0.1979
Vasoactive drugs during ICU stay, n (%)	169 (70.1)	133 (85.3)	36 (49.3)	<0.0001
ECMO during ICU stay, n (%)	52 (21.6)	45 (28.3)	7 (8.6)	0.0004
ECMO at ICU admission, n (%)	44 (18.3)	37 (23.3)	7 (8.6)	0.0049
Days in ECMO, median (IQR)		17 (10–24)	4 (2–8)	0.0008
Enteric colonization, n (%)	129 (53.5)	111 (69.8)	18 (22.2)	<0.0001
Previous carbapenems use, n (%)	40 (16.6)	31 (20.8)	9 (11.5)	0.0993
**Outcomes**	**Total** **(*N* = 241)**	**Superinfection** **(*N* = 159)**	**No Superinfection (*N* = 82)**	***p*-Value**
ICU mortality, n (%)	133 (55.2)	108 (67.9)	25 (30.5)	<0.0001
Hospital mortality, n (%)	145 (60.2)	116 (73.0)	29 (35.4)	<0.0001
ICU LOS, days, median, (IQR)		18 (12–27)	8 (5–11)	<0.0001
Hospital LOS, days, median, (IQR)		25 (18–34)	19 (13–31)	0.0027
Pre-ICU LOS, days, median, (IQR)		3 (1–7)	2 (1–7)	0.3955

List of abbreviations: BMI: Body Mass Index; CPAP: Continuous Positive Airway Pressure; ECMO: Extra-Corporeal Membrane Oxygenation; ICU: intensive care unit; IMV: invasive mechanical ventilation; iNO: inhaled nitric oxide; LOS: length of stay; NIV: non-invasive ventilation; RRT: renal replacement therapy.

**Table 3:** Microorganisms isolated in patients with superinfection.

Isolated Microorganisms	Superinfection 1st Episode	Superinfection 2nd Episode	Total *N* (Overall %)
Carbapenem-resistant *Acinetobacter baumannii*	33	31	64 (24.3)
Carbapenem-resistant *Klebsiella pneumoniae*	17	19	36 (13.7)
*Staphylococcus aureus*			38 (14.4)
Methicillin-susceptible *Staphylococcus aureus* (MSSA)	21	4	25 (9.5)
Methicillin-resistant *Staphylococcus aureus* (MRSA)	10	3	13 (4.9)
*Enterococci*			27 (10.3)
*Enterococcus* faecalis	11	5	16 (6.1)
*Enterococcus* faecium	7	2	9 (3.4)
Vancomicin-resistant (intermediate or total resistance) *Enterococcus faecalis* (VRE)	1		1 (0.4)
Vancomicin-resistant (intermediate or total resistance) *Enterococcus faecium* (VRE)	1		1 (0.4)
Coagulase-negative *Staphylococci* (CoNS)			18 (6.8)
Methicillin-resistant coagulase-negative *Staphylococci (epidermidis, hemolyticus)*	8	4	12 (4.6)
Methicillin-susceptible coagulase-negative *Staphylococci (epidermidis, hemolyticus)*	3	5	6 (2.3)
*Pseudomonas aeruginosa*	8	7	15 (5.7)
*Escherichia coli*	6	2	8 (3.0)
*Serratia marcescens*	5	2	7 (2.7)
*Klebsiella pneumoniae*	4	1	5 (1.9)
*Carbapenem-resistant Pseudomonas aeruginosa*	1	4	5 (1.9)
MDR *Stenotrophomonas maltophilia*	4	1	5 (1.9)
*Klebsiella oxytoca*	3	1	4 (1.5)
*Candida* spp.	2	2	4 (1.5)
*Citrobacter*	1	2	3 (1.1)
*Enterobacter* spp.	10	1	11 (4.2)
*Haemophilus influentiae*	3	0	3 (1.1)
*Streptococcus* spp.	3	0	3 (1.1)
ESBL *Pseudomonas aeruginosa*	1	1	2 (0.8)
*Other microorganisms*	2	0	2 (0.8)
ESBL *Escherichia coli*	1	0	1 (0.4)
*Klebsiella* spp. *(not K. pneumoniae)*	1	0	1 (0.4)
*Listeria monocytogenes*	1	0	1 (0.4)
Total			263 (100)

List of abbreviations: MDR: multidrug-resistant; ESBL: extended-spectrum beta-lactamase.

**Table 4:** Baseline characteristics of patients with and without superinfection by multidrug-resistant (MDR) bacteria.

Variable	MDR Yes (*N* = 97)	MDR No (*N* = 141)	*p*-Value
Age, years, mean ± SD	62.5 ± 10.7	64.17 ± 11.7	0.2195
Gender, male, n (%)	71 (73.2)	108 (76.6)	0.6470
BMI, kg/m^2^, median (IQR)	28.9 (25.9–31.2)	27.8 (25.3–31.5)	0.5982
Lactate concentration, mmol/L, mean ± SD	4.24 ± 22.89	3.3 ± 13.1	0.8534
SOFA at admission, median (IQR)	7 (5–9)	6 (4–9)	0.1598
MuLBSTA at admission, median (IQR)	13 (9–15)	11 (8–13)	0.0010
SAPS II at admission, median (IQR)	48 (39–56)	48 (40–61)	0.1438
SOFA1 (1st ep. superinfection), median (IQR)	8 (7–10)	11 (8–13)	0.6929
Days of superinfection, median (IQR)	7 (4–10)	7 (3–11)	0.8668
Pronation, n (%)	84 (86.6)	88 (62.4)	<0.0001
iNO, n (%)	32 (33.0)	18 (12.8)	0.0003
Vasoactive drugs, n (%)	84 (86.6)	82 (58.2)	<0.0001
Curarization, n (%)	94 (96.9)	106 (75.2)	<0.0001
Steroids, n (%)	78 (80.4)	95 (67.4)	0.0130
ECMO during ICU stay, n (%)	31 (32.0)	20 (14.2)	0.0020
ECMO at ICU admission, n (%)	27 (27.8)	16 (11.3)	0.0018
IMV during ICU stay, n (%)	93 (95.9)	117 (83.0)	<0.0001
Days of IMV, median (IQR)	18 (13–25)	8 (5–16)	<0.0001
Enteric colonization	80 (82.5)	49 (34.8)	<0.0001
- Carbapenem-resistant *Acinetobacter baumannii*	26 (32.5)	10 (20.4)	0.0363
- Carbapenem-resistant *Klebsiella pneumoniae*	50 (62.5)	27 (55.1)	0.0363
Septic shock, 1st ep. superinfection, n (%)	44 (45.4)	26 (18.4)	<0.0001
Previous carbapenems use, n (%)	23 (23.7)	17 (12.1)	0.0208
1st ep. superinfection			0.0763
VAP	70 (72.2)	33 (55.9)	
BSI	26 (26.8)	25 (42.4)	
Other	1 (1.0)	1 (1.7)	
**Outcomes**	**MDR Yes** **(*N* = 97)**	**MDR No** **(*N* = 141)**	***p*-Value**
ICU mortality, n (%)	68 (70.1)	62 (44.0)	<0.0001
Hospital mortality, n (%)	70 (72.2)	72 (51.1)	0.0012
ICU LOS, days, median (IQR)	20 (14–29)	10 (6–17)	<0.0001
Hospital LOS, days, median (IQR)	28 (20–37)	20 (13–31)	<0.0001

List of abbreviations: BMI: Body Mass Index; BSI: bloodstream infection; ECMO: Extra-Corporeal Membrane Oxygenation; HFNC: High-Flow Nasal Cannula; ICU: intensive care unit; IMV: invasive mechanical ventilation; iNO: inhaled nitric oxide; LOS: length of stay; NIV: non-invasive ventilation; SAPS II score: Simplified Acute Physiology Score II; SOFA score: Sequential Organ Failure Assessment score; VAP: ventilator-associated pneumonia.

**Table 5:** Baseline characteristics of patients with and without superinfection by carbapenem-resistant *Klebsiella pneumoniae* (CR-KP).

Variable	CR-KP Yes (*N* = 38)	CR-KP No (*N* = 200)	*p*-Value
Age, years, mean ± SD	60.8 ± 9.3	64.0 ± 11.6	0.0610
Gender, male, n (%)	27 (71.1)	152 (76.0)	0.5410
BMI, kg/m^2^, median (IQR)	30 (26–33)	28 (25–31)	0.2581
Lactate concentration, mmol/L, median ± SD	8.44 ± 37.4	2.80 ± 10.8	0.4252
SOFA at admission, median (IQR)	8 (5–10)	7 (4–9)	0.0967
MuLBSTA at admission, median (IQR)	12 (9–14)	11 (9–13)	0.4208
SAPS II at admission, median (IQR)	48 (38–58)	48 (40–58)	0.6034
SOFA1 (1st ep. superinfection), median (IQR)	8 (7–10)	9 (7–11)	0.1543
Days of superinfection, median (IQR)	8 (5–10)	6 (3–10)	0.2361
Pronation, n (%)	31 (81.6)	141 (70.5)	0.3147
Days of ECMO, median (IQR)	10 (0–24)	0 (0–0)	<0.0001
iNO, n (%)	10 (26.3)	40 (20.0)	0.3815
Vasoactive drugs, n (%)	33 (86.8)	133 (66.5)	0.0236
Curarization, n (%)	35 (92.1)	165 (82.5)	0.2677
ECMO during ICU stay, n (%)	16 (42.1)	35 (17.5)	0.0020
ECMO at ICU admission, n (%)	13 (34.2)	30 (15.0)	0.0099
IMV during ICU stay, n (%)	34 (89.5)	176 (88.0)	0.1412
Days of IMV, median (IQR)	23 (14–34)	12 (6–19)	<0.0001
Days of HFNC, median (IQR)	1 (1–3)	2 (1–4)	0.3075
Rectal colonization	35 (92.1)	94 (47.0)	<0.0001
- Carbapenem-resistant *Acinetobacter baumannii*	2 (5.7)	34 (36.2)	<0.0001
- Carbapenem-resistant *Klebsiella pneumoniae*	33 (94.3)	44 (46.8)	<0.0001
Septic shock, 1st ep. superinfection, n (%)	22 (57.9)	48 (24.0)	0.0002
Previous carbapenems use, n (%)	8 (21.1)	32 (16.0)	0.3385
Empirical antifungal therapy, n (%)	11 (28.9)	42 (21.0)	0.2778
1st ep. superinfection			0.5582
VAP	24 (63.2)	79 (66.9)	
BSI	13 (34.2)	38 (32.2)	
Other	1 (2.6)	1 (0.8)	
**Outcomes**	**KPC Yes** **(*N* = 38)**	**KPC No** **(*N* = 200)**	***p*** **-Value**
ICU mortality, n (%)	24 (63.2)	106 (53.0)	0.2885
Hospital mortality, n (%)	25 (65.8)	117 (58.5)	0.4724
ICU LOS, days, median (IQR)	23 (16–35)	13 (7–20)	<0.0001
Hospital LOS, days, median (IQR)	31 (24–46)	22 (15–32)	<0.0001

List of abbreviations: BMI: Body Mass Index; BSI: bloodstream infection; ECMO: Extra-Corporeal Membrane Oxygenation; HFNC: High-Flow Nasal Cannula; ICU: intensive care unit; IMV: invasive mechanical ventilation; iNO: inhaled nitric oxide; LOS: length of stay; NIV: non-invasive ventilation; SAPS II score: Simplified Acute Physiology Score II; SOFA score: Sequential Organ Failure Assessment score; VAP: ventilator-associated pneumonia.

**Table 6:** Baseline characteristics of patients with and without superinfection by carbapenem-resistant *Acinetobacter baumannii* (CR-AB).

Variable	CR-AB Yes (*N* = 55)	CR-AB No (*N* = 183)	*p*-Value
Age, years, mean ± sd	63.02 ± 11.07	63.60 ± 11.43	0.8546
Gender, male, n (%)	42 (76.4)	137 (74.9)	1
BMI, kg/m^2^, median (IQR)	28 (25–31)	28 (26–31)	0.7579
Lactate concentration, mmol/L, median ± sd	1.79 ± 1.08	4.38 ± 20.86	0.4076
SOFA at admission, median (IQR)	7 (4–8)	7 (4–9)	0.6947
MuLBSTA at admission, median (IQR)	13 (11–15)	11 (8–13)	<0.0001
SAPS II at admission, median (IQR)	48 (36–54)	48 (40–61)	0.1413
SOFA1 (1st ep. superinfection), median (IQR)	9 (7–11)	9 (7–10)	0.2957
Days of superinfection, median (IQR)	6 (4–9)	8 (3–11)	0.3625
Pronation, n (%)	48 (87.3)	124 (67.8)	0.0084
Days of ECMO, median (IQR)	0 (0–11)	0 (0–4)	0.3927
iNO, n (%)	19 (34.5)	31 (16.9)	0.0082
Vasoactive drugs, n (%)	49 (89.1)	117 (63.9)	0.0025
Curarization, n (%)	55 (100)	145 (79.2)	0.0004
ECMO during ICU stay, n (%)	17 (30.9)	34 (18.6)	0.0621
ECMO at ICU admission, n (%)	15 (27.3)	28 (15.3)	0.0705
IMV during ICU stay, n (%)	54 (98.2)	156 (85.2)	0.0017
Days of IMV, median (IQR)	18 (12–23)	11 (6–20)	0.0056
Days of HFNC, median (IQR)	1 (1–3)	2 (1–4)	0.2040
Steroids, days, median (IQR)	10 (8–10)	8 (0–10)	0.0055
Septic shock, 1st ep. superinfection, n (%)	24 (43.6)	46 (25.1)	0.0178
Previous carbapenems use, n (%)	10 (18.2)	30 (16.4)	0.8366
Empirical antifungal therapy, n (%)	12 (21.8)	41 (22.4)	1
1st ep. superinfection			0.2362
VAP	41 (74.5)	62 (61.4)	
BSI	14 (25.5)	37 (36.6)	
Other	0	2 (2.0)	
**Outcomes**	**CR-AB Yes** **(*N* = 55)**	**CR-AB No** **(*N* = 183)**	***p*-Value**
ICU mortality, n (%)	42 (76.4)	88 (48.1)	0.0002
Hospital mortality, n (%)	43 (78.2)	99 (54.1)	0.0016
ICU LOS, days, median (IQR)	18 (14–24)	12 (7–22)	<0.0001
Hospital LOS, days, median (IQR)	26 (19–36)	23 (15–33)	0.0918

List of abbreviations: BMI: Body Mass Index; BSI: bloodstream infection; ECMO: Extra-Corporeal Membrane Oxygenation; HFNC: High-Flow Nasal Cannula; ICU: intensive care unit; IMV: invasive mechanical ventilation; iNO: inhaled nitric oxide; LOS: length of stay; NIV: non-invasive ventilation; SAPS II score: Simplified Acute Physiology Score II; SOFA score: Sequential Organ Failure Assessment score; VAP: ventilator-associated pneumonia.

## Data Availability

The database generated during this study is available on appropriate request by writing to the corresponding author.
